# Structural/functional analysis of the human OXR1 protein: identification of exon 8 as the anti-oxidant encoding function

**DOI:** 10.1186/1471-2199-13-26

**Published:** 2012-08-08

**Authors:** Kenan C Murphy, Michael R Volkert

**Affiliations:** 1 Department of Microbiology and Physiological Systems, University of Massachusetts Medical School, 55 Lake Avenue, Worcester, MA, 01655, USA

## Abstract

**Background:**

The human OXR1 gene belongs to a class of genes with conserved functions that protect cells from reactive oxygen species (ROS). The gene was found using a screen of a human cDNA library by its ability to suppress the spontaneous mutator phenotype of an *E. coli mutH nth* strain. The function of OXR1 is unknown. The human and yeast genes are induced by oxidative stress and targeted to the mitochondria; the yeast gene is required for resistance to hydrogen peroxide. Multiple spliced isoforms are expressed in a variety of human tissues, including brain.

**Results:**

In this report, we use a papillation assay that measures spontaneous mutagenesis of an *E. coli mutM mutY* strain, a host defective for oxidative DNA repair. Papillation frequencies with this strain are dependent upon a G→T transversion in the *lacZ* gene (a mutation known to occur as a result of oxidative damage) and are suppressed by in vivo expression of human OXR1. N-terminal, C-terminal and internal deletions of the OXR1 gene were constructed and tested for suppression of the mutagenic phenotype of the *mutM mutY* strain. We find that the TLDc domain, encoded by the final four exons of the OXR1 gene, is not required for papillation suppression in *E. coli*. Instead, we show that the protein segment encoded by exon 8 of OXR1 is responsible for the suppression of oxidative damage in *E. coli*.

**Conclusion:**

The protein segment encoded by OXR1 exon 8 plays an important role in the anti-oxidative function of the human OXR1 protein. This result suggests that the TLDc domain, found in OXR1 exons 12–16 and common in many proteins with nuclear function, has an alternate (undefined) role other than oxidative repair.

## Background

Respiratory metabolism generates reactive oxygen species (ROS) that can damage many cellular components such as DNA, proteins and lipids [1-3]. These ROS include such molecules as superoxide, singlet oxygen, hydroxyl radicals, and hydrogen peroxide, which can be produced as by-products of aerobic metabolism, oxidoreductase enzymes and metal-catalyzed oxidations. Hydrogen peroxide, while relatively stable, can react with Fe^2+^ via the Fenton reaction to produce hydroxyl radicals [4]. ROS also play a role in cell signaling, where they can be involved in apoptotic processes, transcriptional activation or suppression programs, and cell signaling cascades [5]. An increasing number of human diseases are associated with the damage that ROS cause, including cancer, autoimmune diseases, hypertension and neurodegenerative diseases [3,6-10]. Oxidative damage is also considered a major factor in the mechanisms of aging and age-related diseases such as Parkinson’s and Alzheimer’s [11-13].

There are two modes of action that cells can use to combat the deleterious effects of ROS on cellular constituents: ROS prevention and DNA repair. The first includes molecules that inactivate or inhibit the formation of ROS, thus preventing damage from occurring in the cell. Such detoxification molecules include enzymes like superoxide dismutase, catalase, and glutathione peroxidase [14,15], metabolites such as beta-carotene, lycopene and vitamins A, C and E, and minerals such as selenium and manganese [16]. These systems either prevent ROS from forming or scavenge them before they can cause damage to vital components of the cell. The second means of defense against ROS involves DNA repair enzymes that correct chromosomal damage caused by ROS if they are not inactivated [17]. These enzymes are primarily components of the base excision repair (BER) pathways in both *Escherichia coli* and higher eukaryotes, though nucleotide excision repair (NER), mismatch repair (MMR), and strand break repair mechanisms are also involved in repair of oxidative damage [18,19].

The 8-oxoG modified base is a frequent oxidation product of guanine that is used as a biomarker of oxidative DNA damage [20]. In *E*. *coli*, 8-oxoG pairs with adenine during replication, resulting in a G→T transversion if the lesion is not repaired. The MutM glycosylase (aka Fpg) functions to remove 8-oxoG from DNA, whereas the MutY protein removes the adenine opposite 8-oxoG, giving more time for MutM to work prior to replication [21]. Nth (endonuclease III) and Nei (endonuclease VIII) are two other glycosylases that act principally on the damaged pyrimidines [22]. *E. coli* mutants in most of these genes, either confer sensitivity to exogenous peroxide treatment, and/or display a spontaneous mutator phenotype as a result of their inability to repair spontaneous oxidative damage. Mammalian homologs of these glycosylases have also been described and are an area of intense study [23].

In a previous study using a human cDNA library to identify eukaryotic genes that either prevent or repair oxidative damage, the OXR1 gene was identified by its ability to suppress the spontaneous mutator phenotype of an *E. coli nth mutH* strain [24]. The OXR1 function is highly conserved among eukaryotes, but is not found in prokaryotes. A deletion of the OXR1 gene in *Saccharomyces cerevisiae* causes an increase in sensitivity to hydrogen peroxide [24], and removal of a locus encoding all seven isoforms in *Drosophila melanogaster* results in lethality due to a defect in eclosion (hatching) [25]. Silencing of OXR1 mRNA by 83% sensitized mosquitoes to the harmful effects of hydrogen peroxide in their drinking water. Interestingly, the silencing of OXR1 also resulted in decreased mRNA levels for both catalase and glutathione peroxidase, suggesting that (at least in insects) OXR1 may have a regulatory role in resistance to ROS [26]. A study examining the expression of OXR1 in the mouse retinal cells after exposure to high levels of oxygen showed that OXR1 expression was increased by 3 days exposure, when photocells were resistant to hyperoxia and remained high in the strain that was resistant to hyperoxia. In the sensitive strain of mice, OXR1 levels declined in the retina and the photocells started to degenerate [27]. Transgenic mice expressing the human ApoE-ϵ4 isoform of apolipoprotein ApoE have been characterized as exhibiting structural and functional abnormalities in their mitochondria [28-30]. A recent proteomic analysis of hippocampal cells from these mice identified OXR1 as one of the mitochondrial targeted gene products specifically downregulated following an ischemic insult [31]. By contrast, the hippocampus cells from mice transgenic for ApoE-ϵ3 did not show mitochondrial abnormalities and did not exhibit a reduction for OXR1 transcripts following ischemic insult.

A recent report shows that the Bella mouse (*bel*), identified in a screen for mouse models of human movement disorders, lacks the OXR1 gene. These mice develop normally for 2 weeks following birth, but soon thereafter develop severe ataxia, do not show normal weight gain, and die within a month [32]. The pathological properties of the *bel* mutant mouse were reversed by an OXR1 transgene, confirming that loss of OXR1 was responsible for these neurological defects. Histological analyses of these mice show increased cell death in the granular cell (GC) layer of the cerebellum. These authors also report that OXR1 is overexpressed in amyotrophic lateral sclerosis (ALS) patients and in mouse models of ALS, indicating a possible protective function of OXR1 in this neurodegenerative disorder. Both the human and yeast OXR1 genes are induced by heat and oxidative stress, and their proteins localize to the mitochondria [33]. Localization of the OXR1 protein to mitochondria is significant since this organelle represents a major source of ROS production in the cell.

A bacterial papillation assay for OXR1 activity has been previously described [34,35]. It utilizes a strain containing the *lacZ* cc104 allele [36] in an *E. coli mutM mutY* strain [21]. In this background, the *lacZ* cc104 mutation spontaneously reverts at high frequency to wild type by a GC→ TA transversion (a common mutation found in DNA exposed to oxidizing agents). Overexpression of *mutM* alone completely eliminates GC→TA transversions in this strain, indicating they are primarily due to lesions repaired by the MutM glycosylase, predominantly 8 oxoG [34,35]. Thus, by growth of colonies on minimal lactose plates containing Xgal and IPTG, isolated colonies show high levels of Lac + papillae. The expression of OXR1 suppresses oxidative damage, which can be easily detected by a lower frequency of papillation in this genetic background. In this study, we use this papillation assay to identify which region of the OXR1 protein is important for this suppressive function. Surprisingly, we find that the highly conserved TLDc domain, found in the extreme C-terminal region of most of the OXR1 isoforms, is not required for suppression of mutagenic activity in *E. coli*. Instead, the oxidation resistance function is located in a region of OXR1 encoded by exon 8, in a segment of the gene that encodes a putative helix-turn-helix structural motif.

## Results

### Numbering of the OXR1 protein

**Figure 1:**
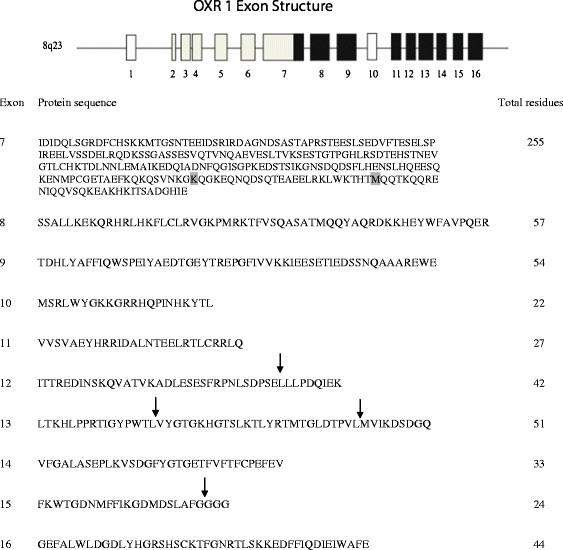
**The human OXR1 gene.** Top: The genomic structure of OXR1 consists of 16 known exons located on chromosome 8q23. Exons, or portions of exons shown in black are present in an oxidation resistance active form of OXR1 previously described [24], whereas exons in white are known to be dispensable for this activity. Exon 10 is found in a variant that begins with exon 10 and includes exons 12 through 15 [43]. Bottom: The protein sequences encoded by OXR1 exons 7 through 16 are listed. The starting points for the series of OXR1 N-terminal truncations were constructed by placing an ATG at the codon prior to position 203 (shaded lysine) of exon 7, using the methionine codon at position 225 (shaded) in exon 7, or by placing an ATG start codon in front of the codons encoding the first residues listed for exons 8 through 13 (except exon 10). The end points for the series of OXR1 C-terminal truncations are denoted by the arrows. Exon 10 is listed here, but was not found in pMV520; it is shown here for completeness.

The original OXR1-producing plasmid pMV520 was identified from a library of human cDNAs cloned into pSE380 [24]. In this construct, the OXR1 DNA sequence starts with codon 200 of exon 7 (shaded lysine in Figure 1) and includes all downstream exons through exon 16, with the exception of exon 10. The start site for OXR1 protein expression from pMV520 is most likely the downstream ATG codon within exon 7 at position 225 (shaded methionine in Figure 1). This proposed N-terminal end for OXR1 encoded by pMV520 is based on the following observations. First, there is an ATG site in the vector (42 base pairs upstream of the OXR1 sequence) that is a potential translational start site for OXR1. There is an ATG site in the vector (42 base pairs upstream of the OXR1 sequence) that is a potential translational start site for OXR1. If used, it would generate an OXR1 protein that has an additional 13 amino acids (encoded by the vector) fused to the N-terminus of OXR1. However, removal of this putative start site (in pMV1248) did not affect the size of the OXR1 protein produced relative to that by encoded by pMV520 (as determined by SDS-PAGE; see section on C-terminal OXR1 deletions described below). This result strongly suggests that the ATG sequence within the vector is not the start site for OXR1 translation in pMV520. Secondly, the vector-encoded start codon in pMV520 is preceded by an in-frame stop codon 15 base pairs upstream and does not have a correctly positioned ribosome binding site preceding it, making it an unlikely start site for translation. Finally, when a construct is made that places an ATG start site in front of codon 200 of exon 7 (pMV1260), an OXR1 product of larger size (relative that produced by pMV520) is observed (data not shown).

These results strongly suggest that OXR1 made from pMV520 starts downstream of the lysine at codon position 200 of exon 7. Thus, the methionine encoded at position 225 of OXR1 exon 7 is designated as the first amino acid residue of “full length” OXR1 protein produced from pMV520 (see Figure 1). This full-length designation is *only* in respect to the region of OXR1 that is responsible for the anti-oxidant activity identified by the papillation assay in these experiments. Splice variants that carry exons not examined here (exons 1–6) may have additional functions, some of which may or may not be involved in oxidative repair. All exons listed in Figure 1, except the first 199 codons of exon 7 and exon 10, are found in the pMV520. Exon 10 is used as an alternative starting point for a splice variant that includes exons 10 and 12 through 16, which has no detectable oxidation antimutator activity (data not shown).

#### OXR1 N-terminal deletions

**Figure 2:**
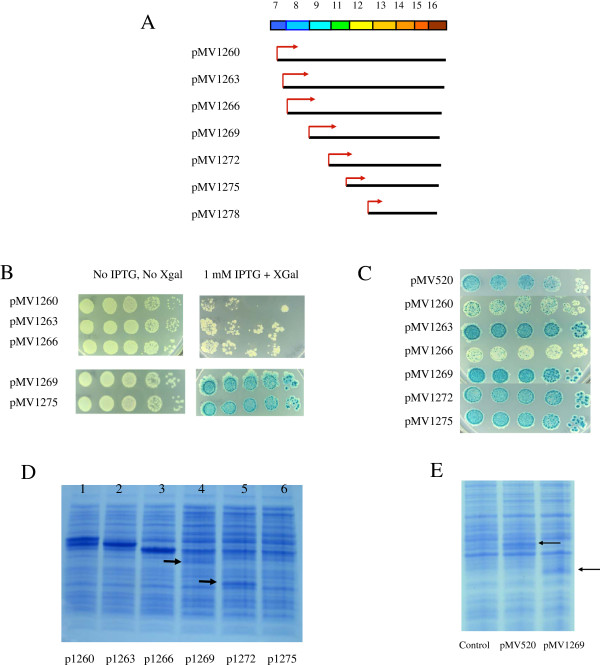
**(A) Diagram of N-terminal deletions of OXR1.** In these assays, the full-length OXR1 construct is represented by both pMV1263, which starts with methionine 225 in exon 7 (the expected start site of OXR1 in pMV520) and pMV1260, which encodes both this species and a slightly larger OXR1 species (by virtue of the presence of an initiating methionine start site inserted prior to codon 200 in exon 7). (**B**) Papillation assay for full-length OXR1 species (pMV1260 and pMV1263) and three N-terminal deletions starting within exon 8 (pMV1266), exon 9 (pMV1269) and exon 12 (pMV1275). Plasmids were expressed in strain MV4709 (*mutM mutY lacZ cc104*) and plated on minimal lactose plates containing 100 μg/ml carbenicillin with and without 1 mM IPTG. Aliquots of 10-fold serial dilutions are plated beginning with undiluted cells on the left. No OXR1 controls containing pBR322 showed patterns that were identical to pMV1269 and p1275 (not shown). (**C**) Papillation assay for full-length OXR1 species (pMV1260 and pMV1263) and three N-terminal deletions starting within exon 8 (pMV1266), exon 9 (pMV1269) and exon 12 (pMV1275). Plasmids were expressed in strain MV4709 (*mutM mutY lacZ cc104*) containing pMS421 (LacI-producer) and plated on minimal lactose plates containing 100 μg/ml carbenicillin, 40 μg/ml streptomycin, 20 μg/ml spectinomycin and 1 mM IPTG. (**D**) SDS-PAGE of extracts containing plasmids expressing OXR1 N-terminal deletions. Plasmid pMV1263 makes the full-length OXR1 species encoded by pMV520, while pMV1260 makes both this species and a slightly larger one that starts at position Lys-200 in exon 7. In addition, the arrows denote positions of observed protein bands for pMV1269 and pMV1272. (**E**) SDS-PAGE comparing full length OXR1 (pMV520) and OXR1 fragment starting at exon 9 (pMV1269). Arrows denote positions of induced protein bands; full length OXR1 is present as a doublet (see text for details).

A series of plasmids containing N-terminal deletions of the OXR1 gene was transformed into strain MV4709 (*mutM mutY*) and tested for suppression of G→T transversions using the papillation assay described above. In these constructs, OXR1 is driven by P_mac_, an artificial promoter designed to express genes under the control of IPTG (see Methods section). The plasmids also encode the *lacI* repressor. As seen in Figure 2B, plasmids pMV1260 and pMV1263 that overexpress full-length OXR1 protein show a reduced plating efficiency upon induction, indicating they are toxic to *E. coli* in the presence of 1 mM IPTG. (In addition, pMV1260 also produces a larger protein that is generated by the transcription of sequences present, but not expressed, in pMV520; see legend to Figure 2A). A reduced plating efficiency was also observed for IPTG induction of cells containing pMV1266, which is missing all of exon 7, and produces an OXR1 N-terminal truncation starting with exon 8 sequences. However, suppression of papillation is still observed among the colonies that do survive containing these three plasmids. On the contrary, OXR1 constructs starting with exons 9 or 12 (pMV1269 and pMV1275) are not toxic to *E. coli* upon IPTG induction and were unable to suppress the papillation phenotype. These results suggest that exon 8 is important for the mutagenic suppressor activity of OXR1, and/or the stability of the OXR1 protein.

As is apparent in Figure 2B, constructs pMV1260, pMV1263 and pMV1266 all show decreased colony forming ability relative to the non-suppressing plasmids in the presence of IPTG. This effect is observed despite the fact that these plasmids encode the *lacI* repressor. The ability of these plasmids to suppress the mutagenic phenotype of MV4709 may be due to the lower viability (or slower growth rate) due to overexpression of a toxic protein. To control the amount of OXR1 deletion fragments produced in vivo, strain MV4709 containing these plasmids were transformed with a compatible plasmid overexpressing the LacI repressor (pMS421), resulting in higher levels of LacI repressor being present (relative to cells expressing *lacI* from only the Oxr1-expressing plasmids). As shown in Figure 2C, this increase in LacI repressor protein expression in vivo had the effect of eliminating the toxicity effect of these plasmids when plated in the presence of 1 mM IPTG, without eliminating the suppressive effects of OXR1 expression on papillation (except pMV1263; discussed below). The papillation assay in this background shows that a construct expressing OXR1 starting at exon 8 (pMV1266) suppresses the mutagenic phenotype of MV4709 without causing toxicity. As before, constructs starting at exons 9, 11 or 12 did not exhibit any suppressor activity.

The lack of papillation suppression by the OXR1 fragment encoded by pMV1269, pMV1272, and pMV1275, might be due to differential expression of the OXR1 deletion mutant proteins. Thus, extracts of cells containing these constructs were run on SDS-PAGE. As seen in Figure 2D, one observes a decrease in OXR1 fragment production upon loss of exon 8 (compare lanes 3 and 4), suggesting the importance of the protein domain encoded by exon 8 in OXR1 stability in *E. coli*. While the decrease in protein production seen with OXR1(9–16) probably explains the loss of toxicity observed with pMV1269 in Figure 2B, we do not believe that this decrease in protein level can explain the inability of OXR1 (9–16) to suppress papillation. This conclusion is based on the observation that similar levels of protein are seen in extracts of cells expressing full length OXR1 from pMV520 (which suppresses papillation) and the OXR1(9–16) fragment from plasmid pMV1269 (which does not suppress papillation – see arrows in Figure 2E). Thus, enough OXR(9–16) fragment is present in cells containing pMV1269 that if it did contain OXR1’s oxidation resistance activity, it would suppress the mutagenic phenotype of MV4709 host cells. Plasmid pMV1272 produces an OXR1 fragment starting at exon 11 and encodes a stable protein but is unable to suppress papillation, while a construct encoding an OXR1 fragment starting at exon 12 (pMV1275) did not produce a stable protein (Figure 2D, lanes 5 & 6, respectively). Finally, a construct expressing only exons 13-16 (pMV1278) also did not show suppression of papillation activity, although it did show a stable protein fragment when extracts were run on SDS-PAGE (data not shown). Overall, these results show that exons 9–16 are not required for the oxidation resistance activity exhibited by full length OXR1. This result was a little surprising, as this region of OXR1 shows the highest homology among comparisons to other OXR1 variants [24] and contains a conserved protein domain found in a number of eukaryotic proteins with nuclear function - the TLDc domain [37].

One other interesting feature is apparent in the results shown in Figure 2C. Under these conditions of limited expression of these OXR1 constructs, the plasmid pMV1263 did not exhibit suppressor activity, even though a plasmid missing an additional 31 residues (pMV1266) did show suppressor activity. This result is not due to a mutation in pMV1263, as sequencing of the promoter and OXR1 region of this construct did not reveal any differences relative to the wild type sequences. Furthermore, this differential suppression is not due to different levels of expression of the OXR1 fragments in these constructs, as both pMV1263 and pMV1266 show similar levels of protein expression on SDS-PAGE gels (see Figure 2D, compare lanes 2 and 3). One possibility for this observation is that the last 25 amino acid residues encoded by exon 7 may contain a control segment for OXR1 activity, which when exposed by removal of exon 7 residues 203–224, results in inhibition of the suppressor activity encoded within exon 8. Note however, that this inhibition can be relieved by further overexpression of this particular construct (when the LacI overproducer is absent - see Figure 2B). Further biochemical and mutational analysis of OXR1 activity within this region will be needed to verify this hypothesis. In addition, this result demonstrates that overproduction of a foreign protein does not in itself induce suppression of the mutagenic phenotype of MV4709.

### C-terminal deletions of OXR1

Carboxy-terminal deletions of OXR1 were constructed to identify what distal exons of OXR1 are important for activity and/or stability of the protein. These plasmids are controlled by the IPTG-inducible P_*trc*_ promoter; the OXR1 sequence starts at position 200 in exon 7 in all the constructs. As described previously, translation from these plasmids most likely initiates with the methionine at position 225 of exon 7. The C-terminal encoding endpoints in these plasmids are found at various points between exons 12 and 16 (see arrows in Figure 1). After transformation into MV4709 containing the LacI-producing plasmid pMS421, the ability of these constructs to suppress mutagenesis was assessed by the papillation assay.

**Figure 3:**
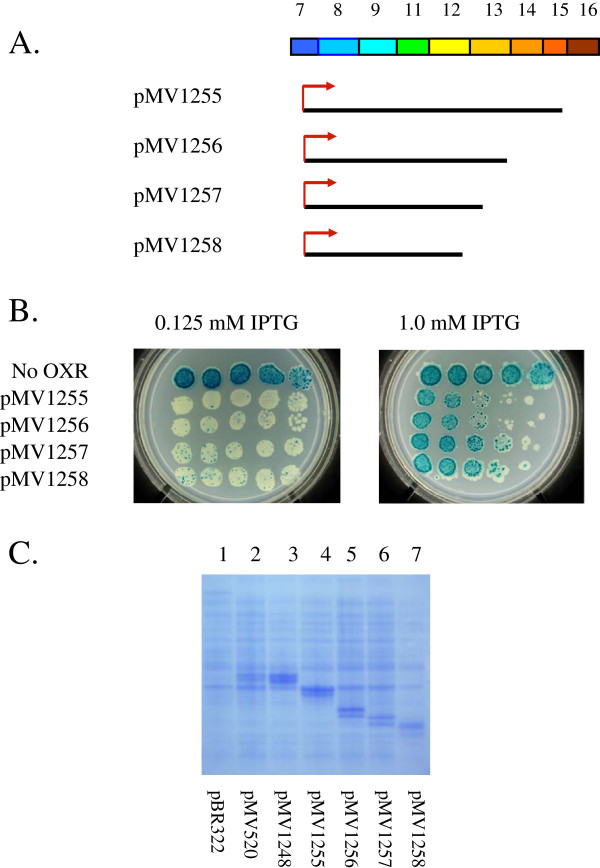
**(A) Diagram of C-terminal deletions of OXR1.** (**B**) Papillation assays with plasmids expressing OXR1 C-terminal deletions in MV4709 containing LacI-producing plasmid pMS421. Dilutions and cell platings were as in Figure 2B using plates containing 0.125 mM and 1.0 mM IPTG on minimal lactose plates containing 100 μg/ml carbenicillin, 40 μg/ml streptomycin and 20 μg/ml spectinomycin. (**C**) SDS-PAGE of extracts containing plasmids expressing OXR1 C-terminal deletions.

As can be seen in Figure 3B, all four of the OXR1 C-terminal deletions were capable of suppressing papillation when plated in the presence of 0.125 mM IPTG. At 1.0 mM IPTG, overexpression of these OXR1 fragments caused toxicity. Interestingly, overexpression of these OXR1 fragments with 1 mM IPTG prevented suppression when high amounts of cells were plated, but still showed suppression of papillation when single colonies were examined (see Figure 3B). This effect was not observed when toxic levels of the N-terminal fragments were plated (see Figure 2B). (The toxicity seen when the C-terminal deletions were plated with 1 mM IPTG in strain MV4709 containing pMV421, but not with the N-terminal deletions described above (Figure 2C), is likely the result of the higher transcriptional activity of the P_trc_ promoter relative to P_mac_.) Thus, removal of OXR1 exons 13–16 (including the TLDc domain) had no effect on the ability of these fragments to suppress papillation (and thus oxidative damage), consistent with the results from the N-terminal deletion constructs described above.

Extracts of MV4709/pMS421 cells expressing these fragments were run on SDS-PAGE to examine the level of proteins produced. As seen in Figure 3C (lane 2), a doublet band is observed for the full length OXR1 expressed from pMV520. Given the close spacing of these bands, the difference in size between them is likely to be about 1–2 kDa. The doublet band of OXR1 is also observed in a second full-length OXR1 construct that promotes higher levels of protein (pMV1248 – see Figure 3C, lane 3) and with all four of the OXR1 C-terminal deletion constructs (see Figure 3C, lanes 4–7). Thus, the most likely explanations for the OXR1 doublet is either (1) a second restart site at the GTG site 13 codons downstream of the ATG start site, or (2) in vivo degradation of 10–20 amino acids from the N-terminus. A construct that initiates from a start codon placed at the beginning of exon 8 encodes a single discrete species (Figure 2D, lane 3). Thus, if the doublet is due to partial degradation, it is limited to sequences encoded within the last 31 residues of exon 7.

### Internal deletions of OXR1 fragment containing OXR1 exons 8–13

**Figure 4:**
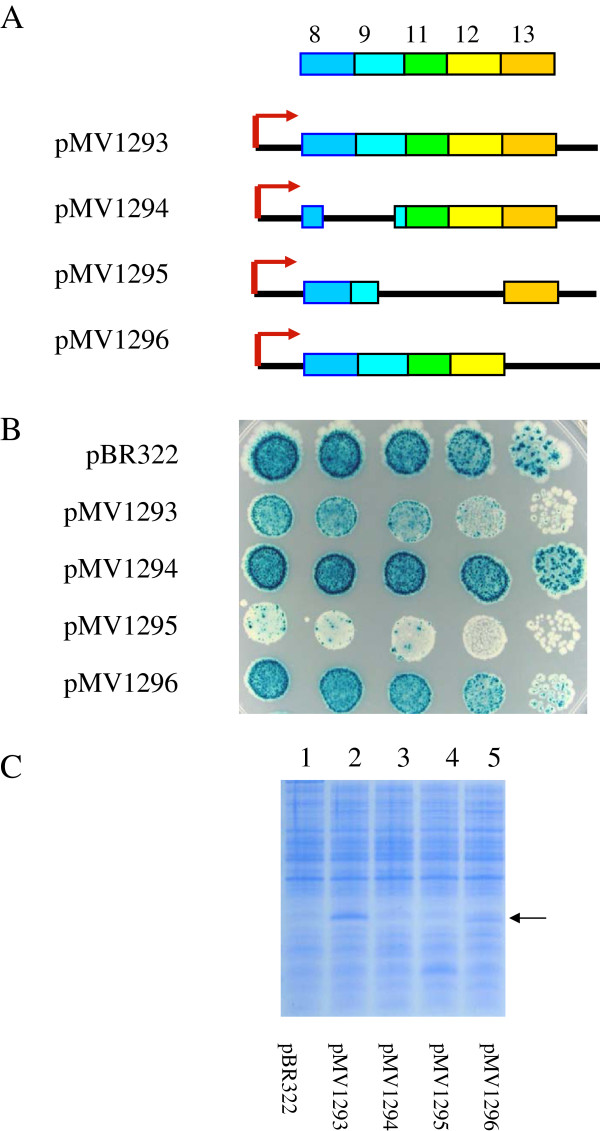
**(A) Diagram of internal-terminal deletions of OXR1 (8–13).** (**B**) Papillation assays with plasmids expressing OXR1 internal deletions in MV4709 containing LacI-producing plasmid pMS421. Dilutions and cell platings were as in Figure 2B using plates containing 1.0 mM IPTG on minimal lactose plates containing 100 μg/ml carbenicillin, 40 μg/ml streptomycin and 20 μg/ml spectinomycin. (**C**) SDS-PAGE of extracts containing plasmids expressing OXR1 internal deletions.

Plasmid pMV1282 encodes OXR1 exons 8, 9, 11, 12 and 13 driven by the P_mac_ promoter (see Methods section). A high level of expression of the OXR1 fragment was observed from extracts of IPTG-induced cells containing this plasmid (data no shown). Overexpression of this OXR1 fragment from this construct was toxic (similar to the N-terminal series of deletions described above). To down-regulate the expression of the OXR1 fragment encoded by pMV1282, rrnB T1 and T2 terminators were removed by digestion with *Sac*I and *Xba*I, filling-in with T4 DNA polymerase and dNTPs, and religating. The resulting plasmid (pMV1293) showed lower amounts of the OXR1 fragment relative to pMV1282 as seen on SDS-PAGE (data not shown). It is presumed that removal of the terminator sequences from the plasmid destabilized the resulting transcript. Plasmid pMV1293 expresses exons 8, 9, 11, 12 and thirty-nine out of the fifty-one residues encoded by exon 13. It suppresses the mutator phenotype of MV4709/pMS421 (*mutM mutY*) when single colonies are examined; see Figure 4B. When the OXR1 fragment from pMV1293 is expressed in MV4709/pMS421, a protein band is observed on SDS-PAGE consistent with the expected size of the OXR1 fragment encoded by these exons (25.6 kDa; see Figure 4C, lane 2).

A series of plasmids containing in-frame internal deletions of pMV1293 were constructed and tested for their ability to suppress the papillation of MV4709/pMS421. Plasmid pMV1294 contains an internal deletion that removes most of exons 8 and 9. This plasmid did not produce a stable band on SDS-PAGE, and not surprisingly, did not suppress papillation (Figure 4B & C). This result suggests that this region of the protein is important for stability of the OXR1 (8–13) fragment, as noted above for the N-terminal deletion plasmid pMV1269. Plasmid pMV1295, which is missing the latter half of exon 9, exons 11 and 12, did suppress papillation and showed a band on SDS-PAGE consistent with the size of the expected fragment (~15 kDa; see Figure 4C, lane 4). In fact, the suppression exhibited by pMV1295 was more efficient than that seen with pMV1293, which contains exons 8–13 (see Figure 4B, compare rows 2 and 4). In this case, papillation suppression was noted when high numbers of cells were plated (see Figure 4B, row 4). The only intact exon in pMV1295 is exon 8, showing again that this domain encodes the active suppressor function. Finally, a plasmid that contains exons 8–12 but is missing all but the first 12 residues of exon 13 (pMV1296), still suppressed papillation within single colonies, but to a lesser extent than pMV1293 (an effect likely due to lower amounts of this protein fragment relative to the OXR1 (8–13); see Figure 4C, compare lanes 2 and 5, see arrow). The most likely explanation for this observation is that a stability determinant of this protein is present in exon 13 and that its presence in pMV1295 stabilizes exon 8, enhancing its activity.

### Internal deletions of full-length OXR1 (8–16)

**Figure 5:**
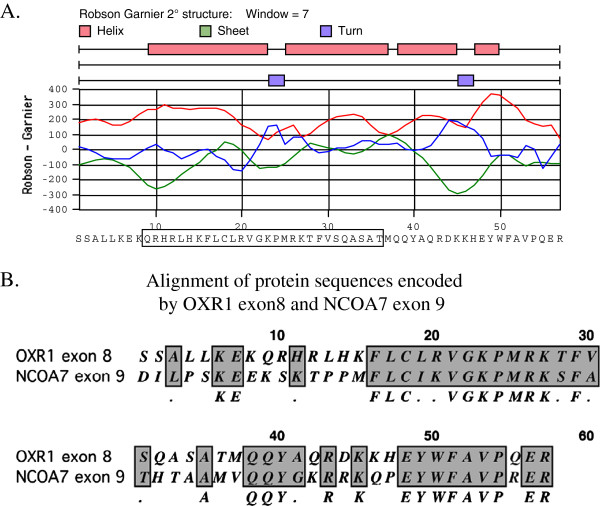
**(A) Secondary structural prediction of the polypeptide encoded by OXR1 exon 8 based on the method of Garnier*et al*[**[38]**].** Boxed region of protein sequence defines the putative larger helix-turn-helix region of the protein. (**B**) ClustelW alignment of the protein sequences encoded by OXR1 exon 8 and NCOA7 exon 9.

**Figure 6:**
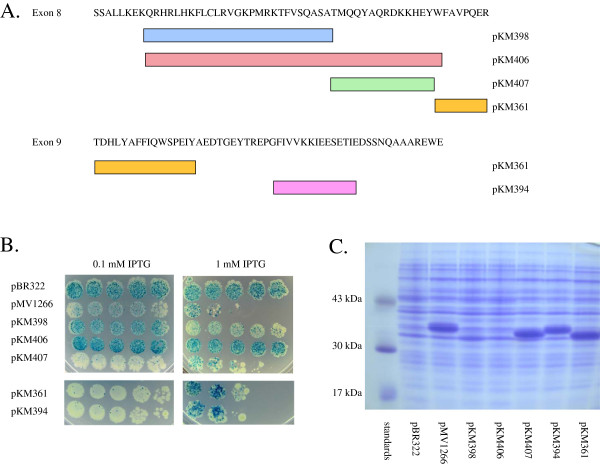
**(A) Diagram of internal deletions of exons 8 and 9 within constructs expressing full length OXR1 proteins (exons 8–16).** Bars represent regions of exon 8 that are missing in the OXR1 deletion mutants. (**B**) Papillation assay for control plasmid (pBR322), an OXR1 encoded by exons 8–16 (pMV1266), and the five internal deletions of OXR1 diagrammed in part A. Plasmids were expressed in strain MV4709 (*mutM mutY lacZ cc104*) containing LacI-producing plasmid pMS421. Dilutions and cell platings were as in Figure 2B using 0.125 mM and 1.0 mM IPTG on minimal lactose plates containing 100 μg/ml carbenicillin, 40 μg/ml streptomycin and 20 μg/ml spectinomycin. (**C**) SDS-PAGE comparing full length OXR1 (pMV1266) and OXR1 deletion mutants described in part A. The expected sizes of the protein fragments from these plasmid are as follows: pMV1266 (38.2 kDa); pKM398 (35.2 kDa); pKM406 (33.1 kDa); pKM407 (36.5 kDa) pKM394 (36.8 kDa); pKM361 (35.5 kDa).

Given the above results suggesting that exon 8 encodes the anti-oxidant activity of OXR1, a Robson-Garnier analysis [38] of the protein secondary structural features of the protein fragment encoded by exon 8 was performed (Figure 5). Highlighted in this analysis is a potential helix-turn-helix segment encoded by residues 9–36 (boxed in Figure 5)A, and a second (smaller) helix-turn-helix segment encoded by residues 38–49. Understanding that protein secondary structural prediction programs are not definitive, we used these predictions as a guide to generate a second set of internal deletions of OXR1, this time starting with the full length construct (containing exons 8–16). Plasmids were constructed that removed each of these regions separately (pKM398 and pKM407) and together (pKM406), as well as deletions in the latter part of exons 8 and 9 (see Figure 6A). Papillation and SDS-PAGE analyses of these constructs are shown in Figure 6B and 6C, respectively.

A plasmid (pKM406) that encodes a protein that is missing both predicted helix-turn-helix structural features failed to suppress papillation on plates containing either 0.1 or 1.0 mM IPTG (Figure 6B, row 4). This result is due to the lack of significant protein production seen with this construct (Figure 6C), again arguing for the presence of a major stability determinant encoded by exon 8. A plasmid (pKM407) that encodes an OXR1 protein that contains the first (larger) predicted helix-turn-helix encoded by exon 8, but not the second one, was fully capable of papillation suppression at 0.1 mM IPTG (see Figure 6B, row 5). The construct also exhibited inhibition of cell growth at higher concentrations of IPTG, as observed with the full length OXR1 protein. A plasmid construct (pKM398) missing the first predicted helix-turn-helix feature, but containing the second one, was also tested. This construct was not able to inhibit papillation at 0.1 mM IPTG, but did so in the presence of higher amounts of IPTG (See Figure 6B). SDS-PAGE analysis of extracts of these cells (Figure 6C) shows that this effect could largely be attributed to the differences in stable protein production from plasmids pKM398 and pKM407 (compare lanes 4 & 6 in Figure 6C), making it hard to assign the supression of mutagenesis function to principally one or the other region in exon 8. Nonetheless, these results show that either predicted helix-turn helix region of exon 8 is sufficient to prevent oxidative damage. In addition, the first predicted helix-turn-helix region of exon 8 encodes the major stability determinant of the OXR1 protein. Finally, a plasmid construct encoding OXR1 deleted of the latter part of exon 8 and the beginning of exon 9 (pKM361), and a plasmid expressing OXR1 deleted of a predicted helix in exon 9 (pKM394), both formed stable proteins that were fully capable of papillation suppression at 0.1 mM IPTG (Figure 6B and C). These results are in agreement with those discussed above for N-terminal deletion mutants that exon 9 is not involved in oxidative damage protection.

2A summary of these experiments is shown in Table 1. It is evident that all the constructs that express a stable form of OXR1 containing exon 8 are fully (or partially) capable of suppressing the oxidative mutator effect exhibited by the tester strain, MV4709 (*mutM mutY*). All other deletions have neither an effect on toxicity (when overexpressed) nor the capability to suppress papillation. Thus, exon 8 encodes the peptide sequences required for the oxidative mutagenesis suppression activity of the human OXR1 protein. Finally, we note that the exon 8-encoded protein sequence contains a cysteine residue at position 18, that might be a reactive residue involved in oxidative damage repair. We tested this prediction by altering Cys-18 of exon 8 to alanine in plasmid pMV1266. However, the C18A mutant was as capable as the wild type OXR1 sequence for suppression of papillation, ruling out a role for this cysteine residue in oxidative damage repair or prevention.

**Table 1:** Papillation results from plasmids expressing OXR1 derivatives

**Plasmid #**	**Description**	**Promoter; terminators**	**Papillation suppression**
pMV520	Exon 7 (200) – Exon 16 (45)	Ptrc	+
C-terminal deletions:	regions present*		
pMV1248	exon 7 (200) – exon 16 (44)	Ptrc	+
pMV1255	exon 7 (200) – exon 15 (20)	Ptrc	+
pMV1256	exon 7 (200) – exon 13 (39)	Ptrc	+
pMV1257	exon 7 (200) – exon 13 (15)	Ptrc	+
pMV1258	exon 7 (200) – exon 12 (31)	Ptrc	+
pMV1293	exon 8 (1) – exon 13 (39)	Pmac	+
N-terminal deletions:	regions present*		
pMV1260	exon 7 (200) – exon 16 (44)	Pmac; rrnT1	+
pMV1263	exon 7 (225) – exon 16 (44)	Pmac; rrnT1	+
pMV1266	exon 8 (1) – exon 16 (44)	Pmac; rrnT1	+
pMV1269	exon 9 (1) – exon 16 (44)	Pmac; rrnT1	-
pMV1272	exon 11 (1) – exon 16 (44)	Pmac; rrnT1	-
pMV1275	exon 12 (1) – exon 16 (44)	Pmac; rrnT1	-
pMV1278	exon 13 (1) – exon 16 (44)	Pmac; rrnT1	-
Internal deletions:	regions deleted**		
pMV1294	Δ exon 8 (14) – exon 11 (11)	Pmac	-
pMV1295	Δ exon 9 (42) – exon 13 (10)	Pmac	+
pMV1296	Δ exon 13 (13–39)	Pmac	+/−
pKM361	Δexon 8 (51) – exon 9 (16)	Pmac; rrnT1	+
pKM394	Δexon 9 (29–42)	Pmac; rrnT1	+
pKM398	Δexon 8 (9–36)	Pmac; rrnT1	+
pKM406	Δexon 8 (9–50)	Pmac; rrnT1	-
pKM407	Δexon 8 (37–50)	Pmac; rrnT1	+

### Quantitative analysis of OXR1 mutation suppression

**Figure 7:**
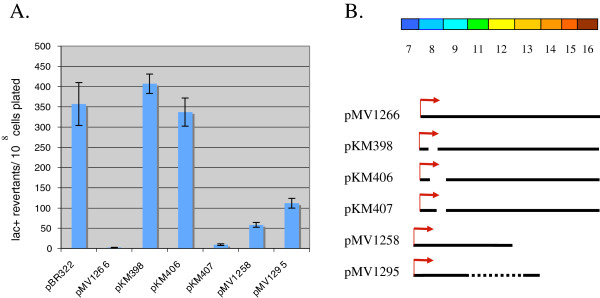
**(A) Lac + reversion frequencies of MV6543 (MV4709 with pMS421) containing various OXR1 fragment-producing plasmids.** Cells containing plasmids were grown overnight as described in the Methods section. All cultures contained 1 mM IPTG, except ones that contained pMV1266 and pKM407, which contained 0.1 mM IPTG (to prevent lethality that results from higher levels of expression of these OXR1 proteins). All cultures grew to saturation (2–4 x 10^9^ cells/ml). The results are reported as the mean of 3–6 determinations for each strain; error bars report the standard error. A *t*-test analysis of the values reported for pKM398 and pKM406 determined that there is no significant difference between the two frequencies reported for these plasmids. (**B**) Diagram of the OXR1 fragment-producing plasmids used in the Lac + reversion assay.

The papillation assay is very sensitive for detection of the mutagenic suppression activity of OXR1. Nonetheless, we preformed a more quantitative analysis on some of the OXR1 fragment-producing constructs described above. Lac^+^ reversion frequencies of MV6543 (*mutM mutY lacZ*cc104) were determined by growing cells overnight and plating on M9 minimal plates containing lactose as the sole carbon source (as described in the Methods section). In the absence of OXR1, MV6543 generated over 350 Lac^+^ revertants per 10^8^ cells plated (see Figure 7A). Expression of an OXR1 fragment, starting with sequences encoded by exon 8 (pMV1266), fully suppressed Lac^+^ reversion frequencies of MV6543 by 99.4%, down to levels exhibited by wild type *E. coli* (Figure 7A; [34]). Plasmid pKM406, which encodes an OXR1 derivative that is largely deleted of exon 8, did not suppress the mutagenic activity of MV6543, consistent with the papillation assay. In large part, this result is likely due to the inability of this construct to produce a stable protein (see Figure 6C). Plasmid pKM398, deleted of exon 8 sequences encoding residues 9–36, also showed no suppression of mutagenic activity in the Lac^+^ reversion assay. This result differs from the papillation assay, where growth of cells containing pKM398 (only in the presence of 1 mM IPTG) did suppress papillation to a degree (see Figure 6B). These different results for pKM398 can be ascribed to either the greater sensitivity of the papillation assay in measuring mutagenesis, or to differences in the residual activity of this OXR1 deletion mutant when grown in liquid culture (in LB) versus M9 minimal plates. Plasmid pKM407, however, deleted of sequences encoding residues 37–50 of exon 8, inhibited mutagenesis by 97% (close to the level exhibited by pMV1266) consistent with the results of the papillation assay described above.

Two other plasmids were also examined in the Lac^+^ reversion assay. Both pMV1258 (encoding a C-terminal deletion of exons 13–16) and pMV1295 (expressing only exon 8 and part of exon 13), which both showed high levels of suppression using the paplliation assay, also showed inhibition of mutagenesis in the Lac^+^ reversion assay to 84% and 69%, respectively, of the level exhibited by MV6543 containing the control plasmid. Again, the lack of full suppression with these plasmids using the Lac^+^ reversion assay can be attributed to the greater sensitivity of the papillation assay, or to differences in OXR1 activity of these mutants when grown in liquid culture versus solid media. Overall, however, the results of the papillation assays and the Lac + reversion frequencies are in good agreement; that is, that exon 8-encoded residues 9–36 play a critical role in the mutagenic suppression capability of the human OXR1 protein.

## Discussion

These results show that OXR1 amino acids 9–36 of exon 8 is a key region responsible for the oxidative antimutator activity of OXR1. This region is also an important stability determinant of the OXR1 protein expression in *E. coli*. While it might be concluded that this region is simply required for stability of OXR1, with the repair function encoded by a different region of OXR1, this supposition is not supported by consideration of results from all the deletion mutants. For instance, the analyses of the C-terminal deletion mutants shown in Figure 3 reveal that exons 13–16 are not required for the anti-oxidation activity exhibited by full length OXR1. This result was a little surprising, as this region of OXR1 shows the highest homology among comparisons to other OXR1 variants [24] and contains a conserved protein domain found in a number of eukaryotic proteins with nuclear function - the TLDc domain [37]. Sequences present in exon 12 can also be ruled out as important for OXR1 activity, as plasmid pMV1292 expresses a stable protein (which includes this region), but does not suppress papillation (Figure 2 C&D). Most revealing however, is the result for plasmid pMV1295, which contains exon 8 as the only intact exon of OXR1 and is completely capable of suppressing papillation (Figure 4). Taken together, the results reported here suggest that exon 8, especially the region encoding a large predicted helix-turn-helix region, is the key determinant for the oxidation resistance function of the OXR1 protein.

The human gene NCOA7 encodes a protein that associates with the estrogen receptor, is targeted to the nucleus, and is highly homologous to OXR1 [24,39]. The gene is expressed in a variety of tissues, but is most highly expressed in neurons. It has been proposed that NCOA7, by binding to the estrogen receptor, might alleviate the oxidative damage that results from estradiol metabolism [34]. Other enzymes involved in the repair of DNA damage have also been identified that bind to the estrogen receptor [40-42] and may play a similar role in protecting DNA from oxidative damage. Durand *et al.*[34] showed that C-terminal fragments of NCOA7 containing the TLDc domain were capable of oxidative damage repair in *E. coli*. Of note, however, is that the fragment of NCOA7 used in that assay also contained most of exon 9, the exon homologous to OXR1 exon 8 examined in this report. An alignment of these two exons is shown in Figure 5B. The C-terminal fragment of NCOA7 that was competent for oxidative repair in *E. coli* started with the methionine at position 15 of exon 9. At this position, the C-terminal fragment of NCOA7 contains the regions most homologous to OXR1 exon 8, the exon described here as being critical for OXR1 oxidative damage suppression. Although not part of the TLDc domain (which in OXR1 starts near the end of sequence encoded in exon 12) a comparison of OXR1 sequences from a variety of organisms shows that exon 8 is highly conserved, especially among higher eukaryotes.

OXR1 and its close homolog NCOA7 are produced in many different forms either as a result of alternative splicing, or alternative transcription start sites. Most of these isoforms include exon 8 of OXR1, or its counterpart (exon 9) of NCOA7, indicating that their antioxidant functions are key features of the gene products. However, there are several isoforms that include only the C-terminal TLDc domain that lack exon 8. The best characterized of these isoforms is the OXR1 C7C variant originally described by Fischer *et al.*[43] and its human counterpart. This variant includes OXR1 exon 10, which is not found in other variants of OXR1. The human form of this variant of OXR1 lacks antioxidant activity, as no suppression of oxidative mutagenesis is seen when this protein is highly expressed in the *E. coli mutM mutY* strain (unpublished observations). This result eliminates the possibility that exon 10 can replace the oxidation resistance function of exon 8. These results suggest that in addition to the conserved oxidation resistance function, these highly conserved proteins may have additional functions that have not been characterized and that this hypothetical function is contained within the highly conserved C-terminal TLDc domain.

Oliver *et al.*[32] have recently reported that loss of mouse OXR1 (mOXR1) resulted in the onset of an ataxia phenotype two weeks after birth in *bel* mice, followed by death within the first month of life. The authors found that the cerebellar granual cell layer (GCL) of these mice were sensitive to hydrogen peroxide more than normal controls, an effect that could be reversed by lentiviral expression of an OXR1 transgene. Notably, using a gene-trap construct, they found that in vivo expression of the C-terminal region of mOXR1 (mOXR1-C), which corresponds to exons 12–16 of hOXR1, was capable of reversing the neurological phenotype and early death of *bel* mouse mutant. In addition, they show that mOXR1-C expression could protect both wild-type and *bel* mutant GCL cells from hydrogen peroxide-induced apoptosis. On the contrary, we find that the C-terminal region of the hOXR1 was neither necessary or sufficient for suppression of oxidative damage in *E. coli*, and instead, that the function required for the antimutator phenotype resides in exon 8, which is present in both full-length and intermediate splice variants of hOXR1.

Combining the results of both studies, a model emerges regarding the different functional characteristics of the mammalian OXR1 protein. The TLDc domain in the C-terminus of mOXR1 is clearly required for a key function of OXR1 that protects cells from oxidative damage, as shown by the study of Oliver *et al.*[32]. These authors go on to show that the mOXR1-C domain can interact with H_2_O_2_ via oxidation of a reactive cysteine (Cys753), suggesting that OXR1’s oxidative antimutator function may be the result of a direct interaction between the protein and reactive oxygen species. However, as the authors noted, the rate constant for this reaction was thousands of times lower relative to antioxidants that work in this manner (such as catalase and peroxiredoxins), leading them to suspect that the TLDc domain of mOXR1 has another type of function, or perhaps operates in a regulatory capacity. A regulatory role of OXR1 has been suggested by Jaramillo-Gutierrez *et al.*[26] who showed that silencing of OXR1 in mosquitoes resulted in lower levels of transcripts coding for enzymes such as catalase and glutathione peroxidase, proteins that act directly in detoxification of ROS. In this study, using our *E. coli* assay, we find that an oxidative antimutator function is not found in the hOXR1 C-terminal region, leading to the greater likelihood that this domain may instead have regulatory role, perhaps after an interaction with certain types of reactive oxygen species. Along these lines, the dispensability of the hOXR1 C-terminal region in our *E. coli* assay is not unexpected if the TLDc domain’s principal role in mammalian cells is to regulate antioxidative operons, an effect not likely reproducible in *E. coli*.

We favor a model of hOXR1 where an antioxidant activity is encoded by exon 8, with the C-terminal domain conferring a regulatory region that responds to ROS. At least in *E. coli*, the proposed C-terminal regulatory region is not required for antioxidant function. Splice variants that include full length or intermediate species of hOXR1 may then reflect the need for both antioxidant activity and antioxidant-regulatory functions, whereas in tissues where only the short C-terminal region of hOXR1 is present may require only the regulatory feature of the OXR1 protein to protect from ROS. The study by Oliver *et al.*[32] showed that the shorter mOXR1-C form is present in the brain at low levels, though it is as highly expressed in the cerebellum as full length mOXR1. Such differences may reflect the need for regulatory capabilities of mOXR1 more so in the cerebellum than in the rest of the brain, where both an oxidative antimutator function and a regulatory region are required. As mentioned in the Introduction, these authors also reported an upregulation of intermediate forms of OXR1 in ALS human biopsy samples, as well as in SOD1 mutant mice (a mouse model of ALS). The intermediate forms of OXR1 contain the upstream oxidative antimutator function we have identified here in exon 8 and may reflect the need for this activity as a protective function in ALS patients. It would be interesting to know if mutations in the hOXR1 gene also play a role in sporadic cases of ALS.

What does OXR1 the exon 8-encoding function do? The region of exon 8 required for oxidation resistance shares homology only with other members of the OXR family of proteins suggesting they have a unique function not shared with other proteins that contribute to oxidative stress resistance. However, it has been observed that OXR1 can suppress mutagenesis in bacteria, indicating it does not require additional human proteins for function and can suppress mutagenesis resulting from mutations in genes inactivating several different types of oxidative lesions [24]. These results make it more likely that OXR1 exon 8-encoded region prevents rather than repairs oxidative damage, since oxidative damage repair enzymes typically recognize only a single, or a set of structurally similar lesions. Such a role for OXR1 as an antioxidant protein is further supported by studies of *oxr1* deletion mutants of yeast, which exhibit elevated levels or ROS, suggesting its function is to reduce ROS levels (Fenton and Volkert, unpublished results).

## Conclusion

The human OXR1 gene exon 8 encodes a function required for the suppression of oxidative damage. This oxidative resistance function is distinct from the highly conserved TLDc domain found in the C-terminal region of OXR1. A model is proposed that OXR1 consist of two separable functions involved in oxidative resistance: a C-terminal domain that has a regulatory role in inducing oxidative resistance functions in response to oxidative damage, and an internal region of the protein encoded by exon 8 that possesses a antimutator “protective” function against reactive oxygen species.

## Methods

### Plasmid constructions

Plasmid pMV520 has been described [24]. C-terminal deletion mutants of OXR1 were generated by cloning PCR-generated fragments of OXR1 into the *Eco*R1 and *Xma*I sites of expression vector pTrc99a. In these plasmids, OXR1 fragments are expressed from the P_trc_ promoter. The endpoints of the OXR1 deletions are indicated by the arrows in Figure 1. Plasmids containing N-terminal deletions of OXR1 were generated by cloning PCR-generated fragments of OXR1 into the *Nde*I and *Sac*I backbone of pTP905 (A. Poteete, unpublished). In these constructs, OXR1 fragments are expressed from  the  IPTG-inducible  P_mac_  promoter  (P  moderate lacUV5-like) [44]. The P_mac_ promoter sequence (TTTAC*ATTGTGAGCGGATAACAAT*ATAAT) contains a *lac* operator (italicized) between the −35 and −10 regions. These OXR1 N-terminal deletion vectors also contain an rrnT1 terminator downstream of the gene fragments, which confers greater stability to transcripts resulting in higher expression levels (unpublished observations). Two of these vectors (pMV1260 and pMV1263) start within the latter part of exon 7; the other plasmids in this series were constructed by adding a start codon in front of the first codon of exons 8, 9, 11, 12 and 13, respectively.

A plasmid pMV1293 was constructed that encoded an OXR1 fragment starting with exon 8 and ending at residue 39 of exon 13. A PCR product (using a derivative of pMV520 as a template) was cloned into the *Nde*I-*Xba*I backbone of pMV1266, resulting in removal of the rrn T1 terminator. A series of internal (in-frame) deletions of pMV1293 were constructed by digestion of the plasmid with restriction enzymes and religation of the resulting backbone. Plasmids pMV1294, pMV1295 and pMV1296 were generated by digestion of pMV1293 with *Cla*I, *Mfe*I and *Nco*I, respectively; the *Cla*I digestion was filled-in by T4 polymerase and dNTPs. The backbone (ori-containing) fragments were religated and transformed into competent *E. coli* cells overexpressing LacI (W3110 lacI^q^). The resulting plasmids contain in-frame deletions of one or more of the OXR1 exons present in pMV1293; the residues *absent* in the OXR1 proteins encoded by these plasmids (relative to pMV1293) are listed in Table 1. Plasmid dilutions of pMV520 were used as a template for all PCR reactions.

A second set of internal deletions within a full length OXR1 protein (defined here as the protein encoded by the exons listed in Table 1, with the exceptions of exons 7 and 10) was generated by λ Red recombineering [45,46]. Cassettes encoding a kanamycin-resistant determinant flanked by *Not*I sites (generated by PCR) were recombined into pMV1266 resulting in various deletions of exons 8 and/or 9. An extra G nucleotide was included in one of the primers, so that following digestion of the modified pMV1266 plasmids with *Not*I and religation of the linearized plasmid, regions of OXR1 containing exon 8 and/or 9 were replaced with GCGGCCGCG. The resulting plasmids thus encoded in-frame deletions of OXR1, where the deleted regions of the OXR1 protein were replaced with 3 alanines. A summary of all the plasmids constructed for this study appears in Table 1. All plasmids modifications constructed in this study were verified by sequencing. Primers sequences used to generate the plasmid constructs are available upon request.

### Mutagenesis assays

Full-length OXR1 protein and various truncations/mutations of OXR1 were expressed in strain MV4709 (*mutM::Tn19 mutY::cat*), which also carries the mutant *lacZ* cc104 allele [47]. This *lacZ* allele reverts to wild type by a GC to TA transversion, a common mutation known to occur during oxidative stress due to the high levels of 8-oxoG formation. Thus, one can follow the spontaneous oxidative damage occurring in this strain by the presence of blue papillae in single white colonies on plates containing Xgal and IPTG [47]. In order to moderate expression of OXR1 and mutant derivatives, host cells also contained the compatible plasmid pMS421 (a pGB2 derivative) that overexpresses the LacI repressor. Fresh cultures of MV4709 (*mutM mutY*) containing pMS421 (MV6543) and plasmids expressing wild type and various deletions of OXR1 were grown from overnight stocks to 2 x 10^8^ cells/ml in LB media containing 100 μg/ml ampicillin, 40 μg/ml spectinomycin and 20 μg/ml streptomycin. Dilutions of the cultures were spot titered on minimal plates containing 0.2% glucose, 1X A salts, 1 mM MgSO4, 0.5 mg/ml phenyl-β-D-galactopyranosie (P-Gal), 50 μg/ml carbenicillin, 40 μg/ml X-gal and various concentrations of IPTG (see figure legends). Mutagenesis was observed by the high density of blue papillae present in single white Lac + colonies; suppression of papillation is indicative of OXR1 activity.

The Lac^+^ reversion assay was performed essentially as described by Poteete [48], with some modifications. Small colonies of MV6543 cells (MV4709 with pMS421-Strep^R^-Spec^R^) containing various OXR1 fragment-producing plasmids (Amp^R^), were picked from LB plates containing ampicillin (100 μg/ml), spectinomycin (20 μg/ml), streptomycin (10 μg/ml), and resuspended in LB containing these same concentrations of drugs, and in addition, either 0.1 mM or 1 mM IPTG (see legend to Figure 7). Between 3–6 colonies were picked for each strain containing an OXR1 fragment-producing plasmid. The cultures were grown overnight at 37^o^C with aeration. Cells were collected by centrifugation and resuspended in 1 volume of 1x M9 salts. Undiluted and diluted samples of cells (between 10^7^ and 10^8^ total) were plated on M9 minimal lactose plates to determine the number of Lac + revertants, and on LB plates to determine the total number of cells plated. M9 minimal plates contained 0.2% lactose and were supplemented with thiamine at a concentration of 5 μg/ml . Lac + reversion frequencies are reported as the number of Lac + revertants per 10^8^ cells plated. To reduce background growth on the minimal lactose plates, 10^9^ scavenger cells, TP889 (ΔlacZ::cat) [48] were plated along with various amounts of the test cultures. The scavenger cells cannot revert to Lac+, but deplete the plates of trace energy sources preventing late-arising colonies from appearing, and in turn, lead to more accurate frequencies of Lac + reversion. The scavenger cells were prepared by growing a 50 ml culture of TP889 in LB overnight, collecting cells by centrifugation, washing once with cold 1x M9 salts and resuspending in 5 ml cold 1x M9 salts; a total of 100 μl of scavenger cells were plated with each of the test strains on M9 minimal lactose plates.

### OXR1 Fragment production in vivo

SDS-polyacrylamide gels were used to assess the presence of stable OXR1 protein fragments. Cultures (5 ml) were grown to 2 x 10^8^ cells/ml in LB containing 100 μg/ml ampicillin, 40 μg/ml spectinomycin and 20 μg/ml streptomycin. IPTG was added to 1 mM concentration and cells were aerated by rolling for 2.5 hours. The cultures were then collected by centrifugation and resuspended in 100 μl of 50 mM Tris–HCl, 100 mM NaCl, 1 mM EDTA, 10% glycerol. Cells suspensions were diluted with an equal volume of H_2_0 and then mixed with an equal amount of 2x SDS buffer. Samples were boiled for 3 minutes, run overnight at 50 volts on 10% polyacrylamide gels using the Tricine system [49] or 12% polyacrylamide using Tris buffers as previously described [50]. Gels were stained with Coomassie Blue. All the protein bands identified in this study as OXR1 full-length protein or proteins fragments were dependent upon the addition of IPTG. Cells not grown in the presence of IPTG showed the same pattern as the pBR322 control. A plasmid derivative of pMV1266 that had a frameshift mutation in the OXR1 gene resulted in the same pattern on these SDS-PAGE gels as pBR322-containing cells.

## Competing interests

The authors declare that they have no competing interests.

## Authors’ contributions

KCM constructed the plasmids and performed the experiments. MRV conceived of the study and provided starting materials. KCM and MRV designed the experiments and wrote the paper; both authors approved the final version.
